# Anti-*Helicobacter pylori* and Anti-Inflammatory Effects and Constituent Analysis of Modified Xiaochaihutang for the Treatment of Chronic Gastritis and Gastric Ulcer

**DOI:** 10.1155/2018/6810369

**Published:** 2018-02-26

**Authors:** Xin Chen, Lijuan Hu, Huanhuan Wu, Wei Liu, Shuhe Chen, Aijun Zhou, Yanwen Liu

**Affiliations:** ^1^Key Laboratory of Traditional Chinese Medicine Resources and Traditional Chinese Medicine Chemistry, Hubei University of Chinese Medicine, Wuhan 430060, China; ^2^Hubei Provincial Hospital of Traditional Chinese Medicine, Wuhan 430060, China; ^3^Dongguan Hospital of Traditional Chinese Medicine, Dongguan 523000, China

## Abstract

Chronic gastritis and gastric ulcers are prevalent throughout the world and are considered to be a global health problem. Modified Xiaochaihutang (MXCHT) prescription is broadly used in traditional medicine hospital for the treatment of gastritis. In order to assess the anti-*Helicobacter pylori (H. pylori)* effect of MXCHT, agar diffusion method* in vitro* and fluid dilution method for the minimal inhibitory concentration (MIC) were established. The anti-inflammatory effects were then evaluated using mouse ear edema model and rat paw edema model. The ethanol-induced gastric ulcer method was employed to verify the gastroprotective effect of active extracts in MXCHT. HPLC-TOF-MS/MS was used for analyzing the possible active constituents after oral administration of effective extracts in ethanol-induced gastric ulcer models. MXCHT and 4 different extracts of the bacterial inhibition diameter and MIC were dramatically decreased compared with control group, showing anti-*Helicobacter pylori* effects. High dose groups of MXCHT, water extract, EtOAc extract, and *n*-BuOH extract displayed significant anti-inflammatory effects in xylene-induced mouse ear edema model and carrageenan-induced rat paw edema model test. MXCHT and all active extracts exhibited gastroprotective activity and prevented gastric lesions induced by ethanol in rats. 4 prototype components and 4 metabolites were identified after oral administration of EtOAc extract. In addition, 6 prototype components and 6 metabolites were identified in *n*-BuOH extract. MXCHT, EtOAc extract, and *n*-BuOH extract demonstrate gastroprotective effects through anti-*Helicobacter pylori* and anti-inflammatory activities. Thus, this prescription may be a suitable natural source for the prevention and treatment of chronic gastritis and gastric ulcers.

## 1. Introduction

Chronic gastritis and gastric ulcer are very prevalent digestive tract diseases throughout all ages in the world and are considered to be a global health problem [1]. Usually they are resulting in an imbalance between the protective factors and aggressive factors in the gastric mucosa [2]. Furthermore, they can be triggered by a range of factors, such as a* Helicobacter pylori (H. pylori)* infection [3], alcohol, stress, and long-term use of nonsteroidal anti-inflammatory drugs (e.g., aspirin, ibuprofen, and naproxen) [4]. Early diagnosis and treatment could enhance patients' well-being and decrease the ultimate risk of gastric cancer; hence, early diagnosis of* H. pylori* infection is of critical importance [5]. Till now, chronic gastritis and gastric ulcer remain a poorly understood entity, with no current effective pharmacological strategies for the management of chronic gastritis and related dyspeptic symptoms [6]. Chronic inflammation plays important roles in the development of various cancers, particularly in digestive organs, including* H. pylori*-associated gastric cancer [7].

*H. pylori* infection is the most common cause of chronic inflammation of the stomach worldwide [8]. The bacterium discovered by Warren and Marshall in 1982 colonizes around half of the world population. All* H. pylori* infected individuals could develop chronic gastritis [7–10].* H. pylori* has a high global prevalence, especially in developing countries [11]. Despite the high prevalence of* H. pylori* infection, it is estimated that 1% of infected people will develop noncardia gastric cancer [12]. Though the oral administration of antibacterial agents, such as amoxicillin, Clarithromycin and metronidazole, is carried out around the world to treat patients infected with* H. pylori*, resistance against these antibacterial agents has been increasing year after year [13, 14]. As a result a great need has arisen to discover and develop new anti-*H. pylori* remedies especially from herbs that not only will eradicate and possibly prevent the organism, but also would have minimal side effects, be easily accessible, and be affordable even for the poor.

Xiaochaihutang (XCHT), a classic Chinese herbal prescription, was first recorded in “Shanghan Lun” two thousand years ago [15, 16]. It consists of seven Chinese common herbs: Chaihu (Radix Bupleuri), Huangqin (Radix Scutellariae), Renshen (Ginseng), Banxia (Pinellia tuber), Radix Glycyrrhizae (Family: Leguminosae; Latin name:* Glycyrrhiza uralensis* Fisch.), Gancao (Radix Glycyrrhizae), Shengjiang (Rhizoma Zingiberis Recens), and Dazao (Fructus Jujubae). Radix Bupleuri is the chief active ingredient of XCHT, and it has been widely used for the treatment of various inflammatory disorders such as chronic hepatitis [17], bronchitis, the common cold, pneumonitis, enterogastritis [18], and depressive disorders in China [19, 20].

In our research, this classic prescription was improved by more than ten years' clinical experience in south cities of China with characters of high efficacy, low recurrence, low side-effect, and low cost. In this modified prescription, Renshen (Ginseng) was replaced by Dangshen (Radix Codonopsis) for cost reduction; furthermore, another four chinense herbs, Huanglian (Rhizoma Coptidis), Ganjiang (Rhizoma Zingiberis), Fuling (Poria), and Baizhu (Rhizoma Atractylodis Macrocephalae), were added for the functions of wind-cold/heat dual-purpose, pungent drugs for dispersion, and bitter drugs for purgation and tonification and purgation in combination and created modified Xiaochaihutang (MXCHT).

This prescription has been used for the treatment of chronic gastritis and gastric ulcer by* H. pylori* infection with high cure rate, which was caused by irregular living habits and fast-paced work environment. To our knowledge, no scientific evidence on treatment of chronic gastritis and gastric ulcer of both the classic XCHT and MXCHT has been provided. Therefore, we conducted this study to confirm the efficacy of MXCHT for chronic inflammation of stomach and to explore its active constituents. Our study is the first to investigate the effective extract for anti-*H. pylori* and anti-inflammatory effect of MXCHT and then identify the main constituents after oral administration of active extract by HPLC-TOF-MS/MS method. Our findings not only elucidate the* in vitro* anti-*H. pylori* and anti-inflammatory effects of MXCHT, but also partially reveal the main active components in blood. We suggest that MXCHT is potentially an effective therapeutic approach for the treatment of chronic gastritis and gastric ulcer by* H. pylori* infection.

## 2. Materials and Methods

### 2.1. Materials and Reagents

MXCHT was composed of 15 g of Radix Bupleuri (Family: Umbelliferae; Latin name:* Bupleurum chinense* DC), 10 g of Radix Scutellariae (Family: Labiatae; Latin name:* Scutellaria baicalensis* Georgi), 10 g of Rhizoma Pinelliae [Family: Araceae; Latin name:* Pinellia ternata* (Thunb.) Breit.], 10 g of Radix Codonopsis [Family: Campanulaceae; Latin name:* Codonopsis pilosula* (Franch.) Nannf.], 6 g of Radix Glycyrrhizae (Family: Leguminosae; Latin name:* Glycyrrhiza uralensis* Fisch.), 30 g of Fructus Jujubae (Family: Rhamnaceae; Latin name:* Ziziphus jujuba* Mill.), 10 g of Rhizoma Zingiberis Recens (Family: Zingiberaceae; Latin name:* Zingiber officinale* Rosc.), 6 g of Rhizoma Zingiberis (Family: Zingiberaceae; Latin name:* Zingiber officinale* Rosc.), 3 g of Rhizoma Coptidis (Family: Ranunculaceae; Latin name:* Coptis chinensis* Franch.), 15 g of Poria [Family: Polyporaceae; Latin name:* Poria cocos* (Schw.) Wolf], and 10 g of Rhizoma Atractylodis Macrocephalae (Family: Compositae; Latin name:* Atractylodes macrocephala* Koidz). The crude drugs were purchased from the Jiuzhoutong Chinese Pharmaceutical Co. Ltd. (Wuhan, China) and were authenticated by Professor Keli Chen (Pharmacy Faculty, Hubei University of Chinese Medicine, China). Voucher specimens were deposited in the Herbarium of Hubei University of Chinese Medicine.

Baicalin (110715-201318), Liquiritin (111610-201106), and Saikosaponin A (110777-201309) were from National Institutes for Food and Drug Control. Baicalein (PS1405-1604) and glycyrrhizic acid ammonium salt (PS0061-0020) were from Push Bio-Technology Company (Chengdu, China). Clarithromycin and aspirin, used as positive controls, were obtained from Yabang Pharmacy (Jiangsu, China) and Original Pharmacy (Shenyang, China), respectively; normal saline solution was obtained from Double-Crane Pharmacy (Beijing, China); *λ*-carrageenan was purchased from Sigma Company; chemical grade reagents were purchased from China National Pharmaceutical Group Corporation.

### 2.2. Preparation of Different Extract from MXCHT

Extracts of MXCHT were prepared by three procedures. One procedure is steam distillation, which was used for obtaining volatile oil; the second one is water extraction, which was operated by macerating several dried herbal mixtures in distilled water for 1 h and then boiling three times (100 g/1200 mL the first time; 100 g/1000 mL the second time; 100 g/800 mL the third time) for 1.5 h each time; the third one is sonic extraction (methanol : HCl = 100 : 1, three times, 0.5 h). After separation with different organic reagents by system solvent method based on either the polarity or the structural properties of constituents, five different extracts were obtained, including volatile oil (0.15%, w/w), water extract (13.3%, w/w), CHCl_3_ extract (0.58%, w/w), EtOAc extract (1.17%, w/w), and *n*-BuOH extract (3.61%, w/w). The methanol extract from Rhizoma Coptidis was mixed with CHCl_3_ extract, because of berberine compounds.

### 2.3. Susceptibility Testing

Susceptibility of* H. pylori* to five extracts was determined by the agar cup diffusion technique.* H. pylori* strain (ATCC43504, Beinuo Life Science, Shanghai) was grown on Columbia agar supplemented with 5% sterile defibrinated sheep blood at 37°C for 72 h under microaerophilic conditions (5% O_2_, 10% CO_2_, and 85% N_2_) and 98% humidity. ATCC43504 suspension was diluted with normal saline solution. Clarithromycin was included as positive control while neat solvent without test compounds was used as negative control.

Full prescription and water extract were diluted in 10 mL distilled water; the other four extracts were prepared by 10 mL dissolved in 1% (v/v) Tween-80% and distilled water. As a precaution not to miss trace amounts of antimicrobials, for preliminary screening, a relatively high concentration of each extract was prepared for test with lower concentration for full prescription. Pure Clarithromycin was dissolved in 15 mL distilled water to obtain 16.67 mg/mL concentration.

The antimicrobial screening was carried out by the agar diffusion method [21]. 15 mL of molten Columbia agar at 50°C, supplemented with 5% sterile defibrinated sheep blood, was seeded with 1 mL of 1 : 100 dilutions of fresh overnight culture of ATCC43504 suspension (approximately 1 × 10^5^ cfu/mL) and thoroughly mixed. The mixture was aseptically poured into Petri dishes and allowed to set. A HB-four Oxford Cup was used to make equidistant wells in the seeded agar. 100 *μ*L of the reconstituted extracts was placed in the wells. The drug-positive control and the negative controls were equally placed in their respective wells. Inoculated plates were incubated at 37°C in an incubator under microaerophilic conditions (5% O_2_, 10% CO_2_, and 85% N_2_) for 3 days after which the diameters of the zones of inhibition (mm) were measured. Each experiment was repeated three times to ensure accuracy and the mean of the diameter of the zones of inhibition was calculated. Controls included use of solvent without test extract, although no antibacterial activity was noted in the solvent employed for the test.

### 2.4. Minimum Inhibitory Concentration (MIC) Determination

The MIC was determined using the agar dilution method as previously described [21]. Different extracts were diluted with Columbia agar culture medium to obtain a series of stock solutions at given concentrations. 1 mL of these solutions and 50 *μ*L of fresh ATCC43504 suspension (approximately 1 × 10^7^ cfu/mL) were added to 2 mL test tube. Positive control was prepared with 1 mL culture medium and 50 *μ*L of fresh ATCC43504 suspension (approximately 1 × 10^7^ cfu/mL); negative control was prepared with 2 mL culture medium. Then, all test tubes were incubated at 37°C for 72 h under microaerophilic conditions. The MIC was determined as the lowest concentration of the antibiotics at which the growth of the inoculum was completely inhibited. All tests were performed in duplicate.

### 2.5. Animals

Kunming mice weighing (18–22 g) and male Wistar mice weighing (190–210 g) were supplied by the Experimental Animal Center of Hubei Province. Animals were maintained on a standardized environmental condition (22 ± 2°C, 12 h light/dark cycle) with free access to food and water and housed as six per cage. All experiments were carried out according to the Regulations of Experimental Animal Administration issued by the State Committee of Science and Technology of China.

### 2.6. Xylene-Induced Mouse Ear Edema Model

To evaluate the anti-inflammatory effect of full prescription and five extracts, a xylene-induced mouse ear edema model was induced with topical application of 20 *μ*L xylene solvent described previously [22, 23]. Shortly thereafter, 112 Kunming mice (18–22 g) were randomly divided into 14 groups of eight each, and each mouse was treated with oral administration for 6 days as follows: Group 1 received 0.9% saline solution; Group 2 received 50 mg of aspirin per kg as a positive control; Groups 3 and 4 received one and two times clinical dosage of full prescription per kg body weight, respectively; Groups 5–14 received one and four times clinical dosage of each extract per kg body weight, respectively. Edema was induced by applying 20 *μ*L of xylene to the inner and outer surface of the right ear. The left ear was considered as control. After the application of xylene with 45 min, the mice were killed under ether anaesthesia and both ears were removed and weighed.

(1) Degree  of  swelling=weight  of  right−weight  of  leftInhibition%=Difference in  ear  weightcontrol−Difference in  ear  weighttestDifference in  ear  weightcontrol×100%.

### 2.7. Carrageenan-Induced Rat Paw Edema Model

To evaluate the antinociceptive effect of full prescription and five extracts, a carrageenan-induced mice paw edema model was induced with topical application of 1%  (v/v) carrageenan (100 *μ*L) described previously [24]. Briefly, 112 male Wistar rats (190–210 g) were randomly divided into 14 groups of eight each, and each mouse was treated with oral administration for 6 days as follows: Group 1 received 0.9% saline solution; Group 2 received 75 mg of aspirin per kg as a positive control; Groups 3 and 4 received one and two times clinical dosage of full prescription per kg body weight, respectively; Groups 5–14 received one and four times clinical dosage of extract per kg body weight, respectively. 100 *μ*L of 1% carrageenan suspension in normal saline was injected into the plantar side of right-hind paw of each rat after oral administration of test samples for 1 h. Paw volume was measured with plethysmometer at 1, 2, 3, 4, and 5 h after carrageenan injection. The degree of swelling was evaluated by the delta volume (*a* − *b*), where *a* and *b* are the volume of the right-hind paw after and before the carrageenan treatment, respectively.

### 2.8. Ethanol-Induced Ulcer

The rats were divided into groups of eight animals each pretreated with vehicle (Veh: water plus 1% tween, 1 mL/kg, p.o.), ranitidine (Cbn: 0.04 g/kg, p.o.), MXCHT (16.5 and 33 g/kg, p.o.), or group for effective extracts (0.75 and 1.5 g/kg, p.o.) 7 days before receiving 0.2 mL of ethanol PA (0.5 mL/100 g) in order to induce gastric ulcer. After 1 h of the ethanol administration, the animals were euthanized. Then, flattened stomach was viewed and its lesion stripes were measured with a Vernier caliper to evaluate the gastric mucosal lesion as follows: stripe length was recorded as the lesion score, and the score would double if the stripe width was over 1 mm [25]. The mean lesion score for each group was expressed as lesion index and the lesion inhibition rate was calculated by the following formula:

(2) $$\begin{array}{c} Lesion\;\;inhibition\%=\frac{\left(the\;\;lesion\;\;index\;\;of\;\;lesion\;\;control\;\;group-the\;\;lesion\;\;index\;\;of\;\;treatment\;\;group\right)}{the\;\;lesion\;\;index\;\;of\;\;lesion\;\;control\;\;group}\times 100\%. \end{array}$$

### 2.9. Plasma Sample Preparation

After final administration of extracts (1.5 mL/100 g) with 0.5 h, blood samples from 6 male Wistar mice as blank and dose group were collected from the suborbital vein into heparinized tubes and centrifuged at 3500 rpm for 10 min. The supernatant was obtained and stored at −20°C. All plasma samples from three mice per group were combined into one sample so as to eliminate the individual variability. 1 mL plasma sample was extracted with protein precipitation with 3 mL acetonitrile. After centrifugation at 12000 rpm for 10 min, supernatant was evaporated to dryness under a gentle stream of nitrogen under 40°C. Then the residue was reconstituted with 200 *μ*L of methanol; 0.6 *μ*L of supernatant was injected into the HPLC-TOF-MS/MS system for analysis. Besides, the blank blood sample was processed at the same way.

### 2.10. Analysis of MXCHT by HPLC-TOF-MS/MS System

About classic Xiaochaihutang (XCHT), 44 components, including Saikosaponin, ginsenoside, Baicalin, and Liquiritin, were identified using the HPLC-MS/MS method described in previous reports [26, 27]. In this research, HPLC-TOF-MS/MS was used to identify the chemical constituents of MXCHT's two active extracts, EtOAc extract and *n*-BuOH extract. The HPLC-TOF-MS/MS system consisted of a Agilent 1260 Infinity high performance liquid chromatography (HPLC) system coupled to a Agilent 6530 Q-TOF mass spectrometer (Agilent Corporation, USA). Data were acquired and processed using Agilent MassHunter qualitative analysis B.07.00 software.

Chromatographic separation was performed on an Agilent Zorbax Eclipse Plus C_18_ RRHD (50 × 2.1 mm, i.d., 1.8 *μ*m) with the column temperature maintained at 30°C, and the detection wavelength was 275 nm. The gradient mobile phase is composed of acetonitrile (a) and 0.1% formic acid in water (b) at a flow rate of 0.4 mL/min. The gradient elution program is shown in Table 1. MS analysis was performed by an electrospray source operating in both positive and negative ion modes with the mass conditions as follows: cone voltage was 25 V, and capillary voltage was 3.0 KV in positive ion mode and 3.0 KV in negative ion mode. Nitrogen was used for the desolvation, and cone gas with a flow rate of 8 L/min at a temperature of 320°C and source temperature was 120°C. Collision gas is argon. MS data were collected in the full scan mode from *m*/*z* 100 to 1000 amu.

### 2.11. Statistical Analysis

Statistical analysis was carried out by SPSS 19.0 software for Windows (SPSS, Inc., Chicago, IL, USA). All values are expressed as mean ± standard error of the mean. Data were analyzed by one-way analysis of variance. If the variance was homogeneity, LSD method was adopted, and Dunnett's T3 was used for heterogeneity. LSD method was used because of homogeneity of variance for mouse ear edema model, 3, 4, and 5 h of rat paw edema model. Dunnett's T3 was used because of heterogeneity of variances for 1 and 2 h of rat paw edema model. The level of significance was set at *P* < 0.05.

## 3. Results and Discussion

### 3.1. Effect of Anti-*H. pylori *of Different Extracts from MXCHT

**Figure 1:**
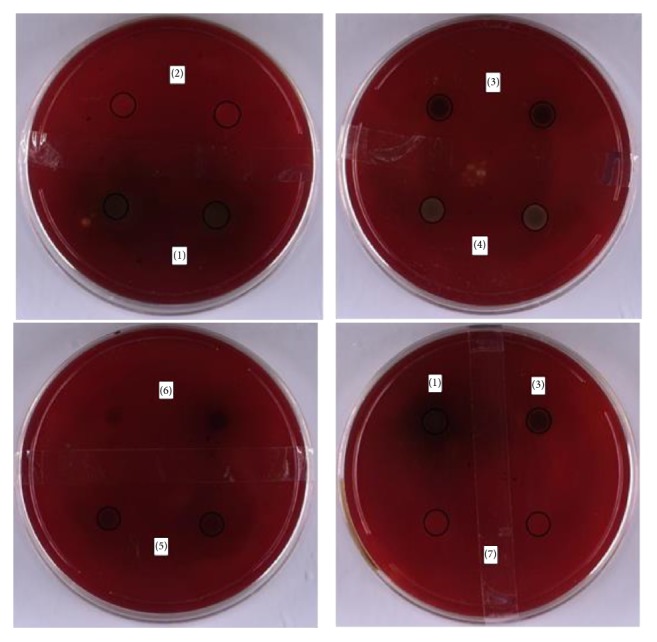
The results of anti-*H. pylori* of different extracts. (1) MXCHT; (2) volatile oil; (3) CHCl_3_ extract; (4) EtOAc extract; (5)* n*-BuOH extract; (6) water extract; (7) positive control.

Full prescription, volatile oil, water extract, CHCl_3_ extract, EtOAc extract, and *n*-BuOH extract were investigated, with Clarithromycin as positive control. In the results of Figure 1 and Table 2, except water extract, full prescription and other four extracts have similar antibacterial susceptibility to Clarithromycin, which demonstrated good activity against* H. pylori.* The reason for the inactivity of water extract may be that the active components of the test sample were unpolar in nature and as such had not been dissolved in water.

### 3.2. Minimum Inhibitory Concentration (MIC) of Different Extracts from MXCHT

The MIC value was determined by adaptive multiple dilution method. The results were shown in Table 3; the bacterial inhibition diameter and MIC of full prescription were 6.90 ± 0.63 mm with 48 mg/mL concentration, the bacterial inhibition diameter and MIC of volatile oils were 7.46 ± 0.47 mm with 7 mg/mL of concentration, the bacterial inhibition diameter and MIC of CHCl_3_ extract were 7.28 ± 0.21 mm with 0.7 mg/mL concentration, the bacterial inhibition diameter and MIC of EtOAc extract were 7.46 ± 0.10 mm with 11 mg/mL concentration, the bacterial inhibition diameter and MIC of *n*-BuOH extract were 7.29 ± 0.10 mm with 12 mg/mL concentration, and the bacterial inhibition diameter of water extract was zero.

### 3.3. Effect of MXCHT and Different Extract on Xylene-Induced Mouse Ear Edema

For xylene-induced mouse ear edema model, compared with control group and high and low dose group of the full prescription and *n*-BuOH extract, high dose group of water extract could very significantly inhibit the mouse ear edema (*P* < 0.01); high dose group of EtOAc extract could significantly inhibit the mouse ear edema (*P* < 0.05). The detailed data was shown in Table 4. The full prescription has obvious anti-inflammatory effect, and main active extracts include water, EtOAc, and *n*-BuOH extracts.

### 3.4. Effect of MXCHT and Different Extract on Carrageenan-Induced Rat Paw Edema

The anti-inflammatory activity of MXCHT and different extract were evaluated on carrageenan-induced paw edema on experimental rats in Table 5. One hour after the oral administration of extract, rats were inflamed with carrageenan. There was gradual increase in edema paw volume in rats in carrageenan treated group showing its maximum value at 4 h. The results showed significant anti-inflammatory activity by all treated groups at 4 h after administration. High and low dose group of the full prescription and *n*-BuOH extract and high dose group of EtOAc extract could very significantly inhibit the rat paw edema (*P* < 0.01); high dose group of water extract could significantly reduce paw swelling of rats (*P* < 0.05). EtOAc extract at 400 mg/kg per body weight, full prescription, and *n*-BuOH extract showed inhibitory effects on carrageenan-induced inflammation during 1–5 h, compared with water extract that only showed effects in the last 1 h. Therefore, the most effective extracts with anti-inflammatory function of MXCHT are EtOAc extract and *n*-BuOH extract.

### 3.5. Effect of MXCHT and Group of Effective Extracts on the Ethanol-Induced Ulcer Models

Total lesion score, lesion index, and lesion inhibition rate were summarized in Table 6. The highest total lesion score and lesion index were found in the control group which had the most serious lesion. Total lesion score and lesion index were dose-dependently decreased in MXCHT or groups of effective extracts. Particularly, lesion index was significantly reduced in rat pretreated with 33 g/kg of MXCHT. The results implied that both MXCHT and groups of effective extracts exerted positive effects against gastric mucosal lesion caused by ethanol.

### 3.6. HPLC-MS/MS Analysis of Plasma Sample after Oral Administration of MXCHT's EtOAc Extract

**Figure 2:**
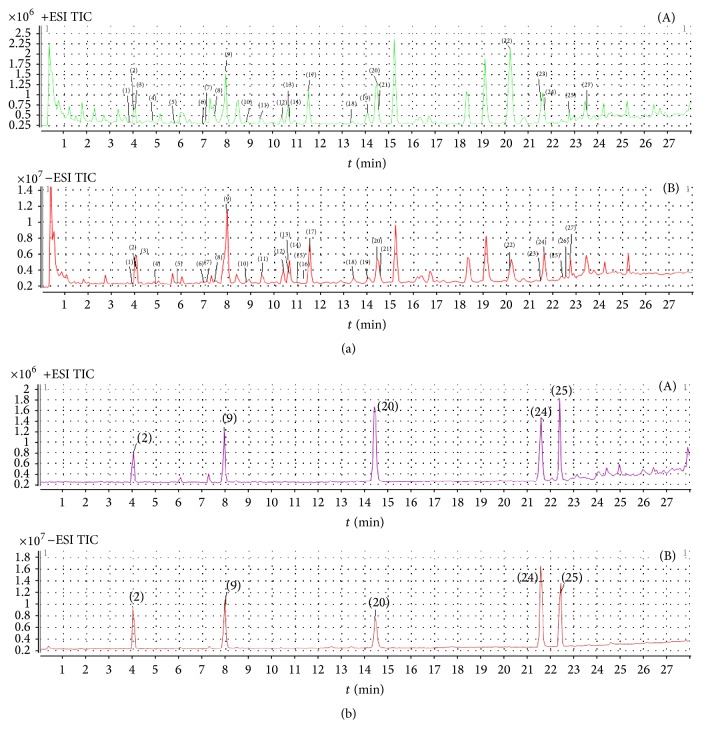
HPLC total ion chromatograms of MXCHT's EtOAc extract (a) and five reference standards (b): Liquiritin, Baicalin, Baicalein, glycyrrhizic acid, and Saikosaponin A ((A) positive ESI; (B) negative ESI).

Based on the results of susceptibility testing, xylene-induced ear edema testing, and carrageenan-induced paw edema testing, plasma samples after oral administration of MXCHT's EtOAc and *n*-butanol extract were analyzed by HPLC-MS/MS method. For EtOAc extract, a total 27 compounds were identified in Figure 2. Corresponding quasimolecular ions and their fragment ions in the MS/MS spectra are summarized in Table 7. By comparing individual peak retention times and the online MS spectra with those of authentic compounds, peaks 2, 9, 20, 24, and 25 were identified as Liquiritin (2), Baicalin (9), Baicalein (20), glycyrrhizic acid (24), and Saikosaponin A (25), respectively, according to standards. The identification of peaks 1, 3–8, 10–19, 21–23, and 26-27 was based on structural information from MS and MS^2^ spectra and comparison of their *m*/*z* values and fragment ions with data from the literature [28–31].

**Figure 3:**
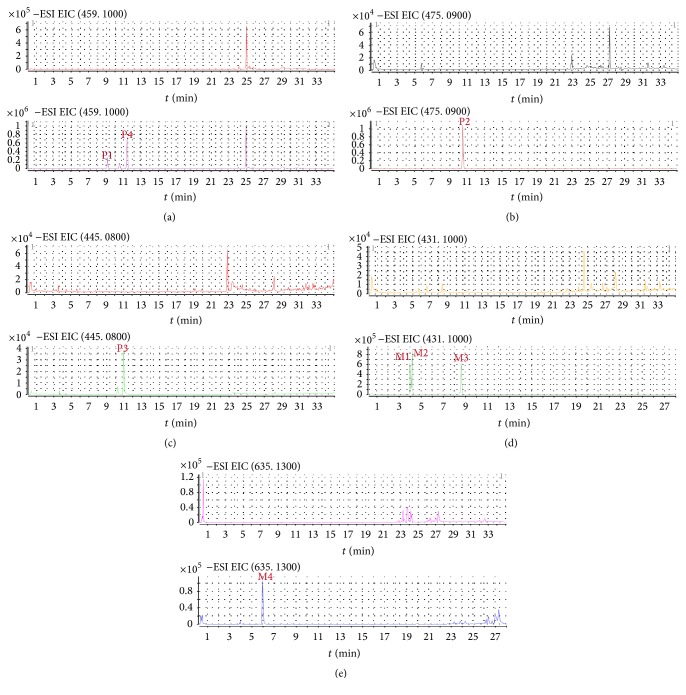
Extracted ion chromatograms of blank plasma and plasma sample 1 h after oral administration of MXCHT's EtOAc extract ((a) P1, P4; (b) P2; (c) P3; (d) M1, M2, and M3; (e) M4).

For dosed plasma of MXCHT's EtOAc extract, 8 compounds, including 4 prototype components and 4 metabolites were identified in Table 8. Extracted ion chromatograms of blank plasma sample and plasma samples after administration of EtOAc extract were shown in Figure 3. There were 4 peaks displayed in the profiles of dosed plasma for MXCHT's EtOAc extract whereas there were no equivalent peaks in the profile of the blank plasma. Thus, these compounds were defined as prototype components. According to the retention times and mass spectra with those of authentic compounds, P1–4 were designated as oroxylin A-7-*O*-glu, trihydroxy-methoxy-glu acid flavone, Baicalein-6-*O*-glu acid, and wogonoside, respectively. The other 4 peaks marked as M1–M4, which only appeared in dosed plasma, were assumed to be exogenous metabolites derived.

M1, M2, and M4 showed the same [M + H]^+^ ion at *m*/*z* 433 and afforded fragment ions at *m*/*z* 257. By referring to the literature data, they were tentatively identified as liquiritigenin-glu acid and isoliquiritigenin-glu acid. In addition, M1 and M2 exhibited the same [M + H]^+^ ions at *m*/*z* 433, and they also displayed the identical fragment ions at *m*/*z* 257, respectively, whereas the retention times of them were different. They were tentatively identified as the isomers of liquiritigenin-glu acid.

In previous studies on the metabolism, Baicalin with a medicinal herb that has been used since ancient times to treat inflammation, fever, and allergic diseases underwent two main metabolic pathways including glucuronidation and methylation [32]. Following the result after administration, the metabolic pathway of wogonoside to wogonin-diglu acid should be glucuronidation. Furthermore,* in vivo* Liquiritin of rats underwent extensive metabolism of phases I and II; initially liquiritigenin was obtained by taking off glucosyl group from Liquiritin, after intestinal mucosa absorption; the phase II conjugation reaction led to the formation of monoglucuronoconjugates, sulfoconjugates, and methyl conjugate under phase II enzymes. These researches were similar to the result in serum pharmacochemistry, which revealed the possible metabolic pathway. This work provides new data about the metabolism of liquiritigenin and shows the interest value of using various experimental models in metabolic studies.

### 3.7. HPLC-MS/MS Analysis of Plasma Sample after Oral Administration of MXCHT's* n*-BuOH Extract

**Figure 4:**
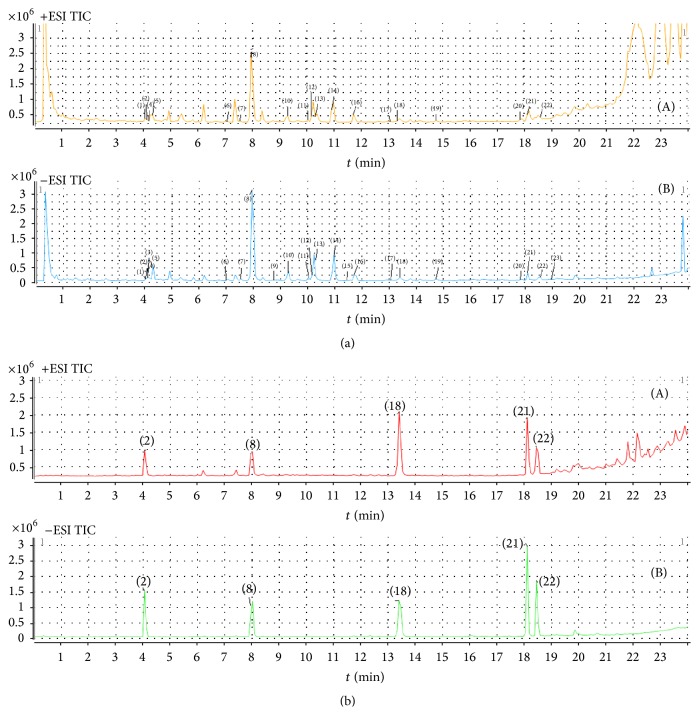
HPLC total ion chromatograms of MXCHT's *n*-BuOH extract (a) and five reference standards (b): Liquiritin, Baicalin, Baicalein, glycyrrhizic acid, and Saikosaponin A ((A) positive ESI; (B) negative ESI).

For *n*-BuOH extract, a total 24 compounds were identified in Figure 4. Corresponding quasimolecular ions and their fragment ions in the MS/MS spectra are summarized in Table 9. By comparing individual peak retention times and the online MS spectra with those of authentic compounds, peaks (2), (8), (18), (22), and (23) were identified as Liquiritin (2), Baicalin (8), Baicalein (18), glycyrrhizic acid (22), and Saikosaponin A (23), respectively, according to standards. The identification of peaks 1, 3–7, 9–17, 19–21, and 24 was based on structural information from MS and MS2 spectra and comparison of their *m*/*z* values and fragment ions with data from the literature [28–31].

**Figure 5:**
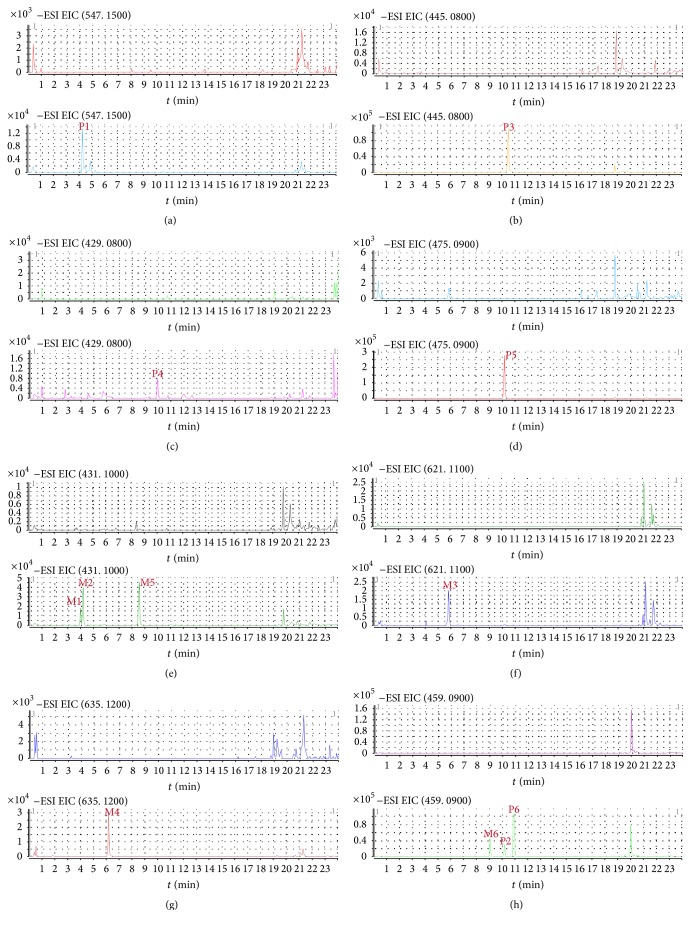
Extracted ion chromatograms of blank plasma and plasma sample 1 h after oral administration of MXCHT's *n*-BuOH extract ((a) P1; (b) P3; (c) P4; (d) P5; (e) M1, M2, and M5; (f) M3; (g) M4; (h) M6, P2, and P6).

For dosed plasma of MXCHT's *n*-BuOH extract, 12 compounds, including 6 prototype components and 6 metabolites were identified in Table 10. Extracted ion chromatograms of blank plasma sample and plasma samples after administration of EtOAc extract were shown in Figure 5.

There were 6 peaks displayed in the profiles of dosed plasma for MXCHT's *n*-BuOH extract whereas there were no equivalent peaks in the profile of the blank plasma. Thus, these compounds were defined as prototype components. According to the retention times and mass spectra with those of authentic compounds, P1–6 were designated as chrysin-6-C-glu-8-C-ara, oroxylin A-7-*O*-glu acid, Baicalein-5-*O*-glu acid, chrysin-glu acid, trihydroxy-methoxy-glu acid flavone, and wogonoside, respectively. The other 6 peaks marked as M1–M6, which only appeared in dosed plasma, were assumed to be exogenous metabolites derived, including liquiritigenin-glu acid, isoliquiritigenin-glu acid, Baicalein-diglu acid, wogonin-5-*O*-glu acid, and wogonin-diglu acid, respectively.

In this work, Saikosaponin A was not detected after oral administration; even the main constituents were saponins. In previous study, the transport form of Saikosaponin A from apical to basolateral was similar to the transport form basolateral to apical. So, the main mechanism of Saikosaponin A intestinal absorption is passive transference [33]. Hence, it was not possible to keep prototype in plasma after oral administration of *n*-BuOH extract.

## 4. Conclusions

Traditional Chinese medicines (TCMs) are natural therapeutic remedies used in China and the Chinese community worldwide for thousands of years, and it is widely accepted that multiple constituents are responsible for their bioactivities. However, due to the complexity of the chemical compositions of TCMs, the bioactive compounds and the therapeutic mechanisms of most TCMs are still unknown up to now. This is the first report that MXCHT was used in therapy of chronic gastritis caused by the stressed life and work in the city. Furthermore, the present results can conclude that EtOAc and *n*-BuOH extract of MXCHT have potential anti-*H. pylori* and anti-inflammatory activities. In addition, the identification and elucidation of active constituents in plasma after oral administration of EtOAc and *n*-BuOH extract provide essential data for the pharmacodynamic profile, which contribute to clarifying the therapeutic basis of MXCHT and facilitating its clinical usage with scientific support. Altogether, the current findings suggest that MXCHT may be a potential candidate as a new therapy for chronic gastritis.

## Figures and Tables

**Table 1:** Gradient elution program of HPLC-TOF-MS/MS method.

Time/min	*a*%	*b*%
0	10	90
0.5	13	87
3.4	17	83
20	32	68
35	100	0

**Table 2:** Antibacterial susceptibility of *H. pylori* to MXCHT and different extracts.

Groups	Concentration of extracts (mg/mL)	Diameter of the zones of inhibition (mm) ± SEM
Positive control	16.7	7.60 ± 0.13
Full prescription	766	6.90 ± 0.63
Volatile oil	5.6	7.46 ± 0.47
Water extract	692	-
CHCl_3_ extract	23	7.28 ± 0.21
EtOAc extract	89	7.46 ± 0.10
*n*-BuOH extract	184	7.29 ± 0.10

Results are an average of duplicate experiments. - = no activity (resistant); diameter of cork borer = 8 mm.

**Table 3:** Minimum inhibitory concentration (MIC) for MXCHT and different extracts on ATCC43504.

Groups	Ratio of dilution								MIC to extracts (mg/mL)
	1 (1 : 1)	2 (1 : 2)	3 (1 : 4)	4 (1 : 8)	5 (1 : 16)	6 (1 : 32)	7 (1 : 64)	8 (1 : 128)	
Positive control	+	+	+	+	+	+	+	+	/
Negative control	−	−	−	−	−	−	−	−	/
Full prescription	−	−	−	−	−	+	+	+	48
Volatile oil	−	−	−	−	+	+	+	+	7
Water extract	+	+	+	+	+	+	+	+	/
CHCl_3_ extract	−	−	−	−	−	−	+	+	0.7
EtOAc extract	−	−	−	−	+	+	+	+	11
*n*-BuOH extract	−	−	−	−	−	+	+	+	12

Results are an average of duplicate experiments. + = turbidity; − = clarity.

**Table 4:** Effect of MXCHT and different extracts on xylene-induced mouse ear edema (*n* = 8, *x* ± *s*).

Groups/dose (mg/kg)	Increase in ear weight (mg)	Inhibition (%)
Model control	7.60 ± 1.70	/
Positive control-50	4.75 ± 1.37^b^	37.5
Full prescription-230	5.56 ± 1.88^b^	26.8
Full prescription-460	4.91 ± 1.38^b^	35.4
Volatile oil-15	7.61 ± 1.44	−0.1
Volatile oil-60	6.42 ± 1.33	15.5
Water extract-15	6.26 ± 2.13	17.6
Water extract-60	4.82 ± 0.93^b^	36.6
CHCl3 extract-15	6.21 ± 1.93	18.3
CHCl3 extract-60	6.95 ± 1.97	8.6
EtOAc extract-15	6.98 ± 1.27	8.2
EtOAc extract-60	5.64 ± 1.17^a^	25.8
*n*-BuOH extract-15	5.06 ± 1.02^b^	33.4
*n*-BuOH extract-60	4.81 ± 1.26^b^	36.7

Values are expressed as mean ± SEM. *n* = 5; ^a^*P* < 0.05 compared with control (ANOVA followed by Dunnett's *t*-test); ^b^*P* < 0.01 compared with control (ANOVA followed by Dunnett's *t*-test).

**Table 5:** Effect of MXCHT and different extracts on carrageenin-induced rat paw edema. (*n* = 8, *x* ± *s*).

Groups/dose (mg/kg)	Change of paw edema volume in mL					
	0 h	1 h	2 h	3 h	4 h	5 h
Model control	0.99 ± 0.045	0.298 ± 0.068	0.385 ± 0.036	0.472 ± 0.037	0.521 ± 0.033	0.526 ± 0.056
Positive control-75	1.02 ± 0.055	0.211 ± 0.070^a^ (29.19)	0.306 ± 0.072^a^ (20.52)	0.352 ± 0.066^b^ (25.42)	0.371 ± 0.070^b^ (28.79)	0.424 ± 0.072^b^ (19.39)
Full prescription-330	1.06 ± 0.032	0.221 ± 0.038^a^ (25.84)	0.340 ± 0.037	0.378 ± 0.059^b^ (19.92)	0.415 ± 0.072^b^ (20.34)	0.454 ± 0.038^b^ (13.69)
Full prescription-660	1.11 ± 0.081	0.211 ± 0.071^a^ (29.19)	0.316 ± 0.096^a^ (17.92)	0.359 ± 0.111^b^ (23.94)	0.390 ± 0.095^b^ (25.14)	0.400 ± 0.091^b^ (23.95)
Volatile oil-100	1.01 ± 0.062	0.265 ± 0.112	0.352 ± 0.081	0.435 ± 0.078	0.474 ± 0.046	0.487 ± 0.047
Volatile oil-400	1.01 ± 0.079	0.312 ± 0.127	0.428 ± 0.091	0.510 ± 0.069	0.529 ± 0.067	0.544 ± 0.076
Water extract-100	1.07 ± 0.036	0.324 ± 0.042	0.402 ± 0.026	0.461 ± 0.033	0.508 ± 0.034	0.459 ± 0.039^a^ (12.73)
Water extract-400	0.93 ± 0.073	0.224 ± 0.060^a^ (24.83)	0.428 ± 0.049	0.469 ± 0.053	0.458 ± 0.059^a^ (12.09)	0.466 ± 0.057
CHCl_3_ extract-100	1.03 ± 0.086	0.324 ± 0.090	0.390 ± 0.091	0.474 ± 0.082	0.505 ± 0.062	0.494 ± 0.091
CHCl_3_ extract-400	0.99 ± 0.05	0.279 ± 0.057	0.414 ± 0.041	0.456 ± 0.043	0.489 ± 0.072	0.471 ± 0.039
EtOAc extract-100	1.01 ± 0.05	0.29 ± 0.029	0.420 ± 0.039	0.449 ± 0.054	0.480 ± 0.065	0.494 ± 0.059
EtOAc extract-400	1.10 ± 0.093	0.208 ± 0.079^a^ (30.20)	0.301 ± 0.032^a^ (21.82)	0.386 ± 0.072^a^ (18.22)	0.406 ± 0.061^b^ (22.07)	0.399 ± 0.067^b^ (24.14)
*n*-BuOH extract-100	1.06 ± 0.044	0.191 ± 0.059^a^ (35.91)	0.345 ± 0.049^a^ (10.39)	0.415 ± 0.057	0.431 ± 0.057^b^ (17.27)	0.439 ± 0.07^b^ (16.54)
*n*-BuOH extract-400	1.09 ± 0.041	0.199 ± 0.040^a^ (33.22)	0.308 ± 0.058^a^ (20.00)	0.388 ± 0.073^a^ (17.80)	0.436 ± 0.063^b^ (16.31)	0.453 ± 0.052^a^ (13.88)

Values are expressed as mean ± SEM. *n* = 5; ^a^*P* < 0.05 compared with control (ANOVA followed by Dunnett's *t*-test). ^b^*P* < 0.01 compared with control (ANOVA followed by Dunnett's *t*-test). Each value in parenthesis indicates the percentage inhibition.

**Table 6:** Effect of MXCHT and group of effective extracts on gastric mucosal lesion induced by ethanol (*n* = 8, mean ± SD).

Groups	Dose (g/kg)	Ulcer index/mm	Ulcer inhibition (%)
Control	-	11.79 ± 5.95	-
Ranitidine	0.04	9.27 ± 3.71	21.44
High MXCHT	33	1.62 ± 1.85^a,b^	86.23
Low MXCHT	16.5	3.13 ± 2.62^a,b^	73.44
High group of effective extracts	1.5	2.44 ± 2.35^a,b^	79.33
Low group of effective extracts	0.75	3.10 ± 2.66^a,b^	73.76

^a^ *P* < 0.01 compared with control (ANOVA followed by Dunnett's *t*-test); ^b^*P* < 0.01 compared with ranitidine (ANOVA followed by Dunnett's *t*-test).

**Table 7:** Chemical components of MXCHT's EtOAc extract.

Number	*t*_*R*_/min	MS data in ESI^+^	MS data in ESI^−^	Identification
(1)	3.879	419.14, 257.08	579.17, 417.12	Liquiritin-glu
(2)	4.026	441.12, 436.16, 257.08	417.12, 255.07	Liquiritin^*∗*^
(3)	4.093	419.14, 436.16, 257.07	417.12, 255.07	Isoliquiritin
(4)	4.832	549.16, 531.15, 483.13, 429.10, 393.10	547.15, 529.15, 457.12, 427.12, 337.07	Chrysin-6-C-glu-8-C-ara
(5)	5.772	417.12	415.11, 295.06	Chrysin-8-C-glu
(6)	6.982	477.11, 499.07, 301.14	475.09, 299.06	Trihydroxy-methoxy-glu acid flavone
(7)	7.116	257.08	517.14, 417.12, 255.07	Isoliquiritin apioside
(8)	7.519	431.14, 269.08	429.13	Ononin
(9)	7.922	447.10, 469.07, 271.06	445.08, 269.05	Baicalin^*∗*^
(10)	8.795	449.11, 273.08	447.10, 271.06	5,6-dihydroxy-7-*O*-glu acid flavanone
(11)	9.400	447.09, 469.08, 271.06	445.08, 269.05	Baicalein-5-*O*-glu acid
(12)	10.340	431.09, 255.07	429.09, 253.05	Chrysin-glu acid
(13)	10.609	461.11, 285.08	459.10, 283.06, 175.02, 113.02	Oroxylin A-7-*O*-glu acid
(14)	10.659	477.11, 301.07	475.09, 299.06	Trihydroxy-methoxy-glu acid flavone
(15)	11.012	-	445.08, 269.05	Baicalein-6-*O*-glu acid
(16)	11.146	-	445.08, 283.27	Oroxylin A-7-*O*-glu
(17)	11.583	461.11, 285.08	459.10, 283.06, 268.04	Wogonoside
(18)	13.397	271.06, 169.01	269.05, 197.06	Isowogonin
(19)	14.035	301.07, 286.05, 184.00	299.06	Trihydroxy-methoxy flavone
(20)	14.438	271.06, 253.05, 169.01	269.05	Baicalein^*∗*^
(21)	14.572	331.08	329.07	Trihydroxy-dimethoxy flavone
(22)	20.215	285.08, 270.05	283.06, 268.08	Wogonin
(23)	21.492	285.08, 270.05	283.06, 268.04	Oroxylin A
(24)	21.626	823.42, 647.38, 471.35, 453.34	821.40	Glycyrrhizic acid^*∗*^
(25)	22.432	803.46, 763.47, 745.46, 619.42, 601.41, 455.36, 437.35	825.47, 799.46	Saikosaponin A^*∗*^
(26)	22.566	-	807.42	Licoricesaponin B_2_
(27)	22.768	803.46, 763.47, 745.46, 619.42, 601.41, 455.36, 437.35	825.47, 799.46	Saikosaponin B_1_

ESI, electrospray ionization source; MS, mass spectrometry; glu acid, glucuronic acid. ^*∗*^Identified with reference standards.

**Table 8:** The prototype components and metabolites detected from plasma of mice after administration of MXCHT's EtOAc extract.

Number	*t*_*R*_/min	MS data in ESI^+^	MS data in ESI^−^	Identification
M1	4.025	433.12, 257.08	431.10, 255.07	Liquiritigenin-glu acid
M2	4.227	433.11, 257.08	431.10, 255.06	Liquiritigenin-glu acid
M3	6.110	637.14, 461.11, 285.08	635.13, 459.09	Wogonin-diglu acid
M4	8.587	433.11, 257.08	431.10, 255.06	Isoliquiritigenin-glu acid
P1	9.267	461.12, 483.09, 285.08	459.10, 283.06, 113.02	Oroxylin A-7-*O*-glu
P2	10.544	477.11, 301.07	475.09, 299.06	Trihydroxy-methoxy-glu acid flavone
P3	11.014	447.09, 469.07, 271.06	445.08, 269,05	Baicalein-6-*O*-glu acid
P4	11.484	461.11, 483.09, 285.08	459.10, 283.06	Wogonoside

ESI, electrospray ionization source; MS, mass spectrometry; P, prototype components; M, metabolites; glu acid, glucuronic acid.

**Table 9:** Chemical components of MXCHT's *n*-BuOH extract.

Number	*t*_*R*_/min	MS data in ESI^+^	MS data in ESI^−^	Identification
(1)	4.119	419.14, 257.08	547.15, 457.12, 427.11, 367.08, 337.07	Chrysin-6-C-ara-8-C-glu
(2)	4.135	441.12, 436.16, 257.08	417.12, 255.07	Liquiritin^*∗*^
(3)	4.186	419.13, 257.08	549.16, 417.12, 255.07	Liquiritigenin apioside
(4)	4.202	419.14, 436.16, 257.07	417.12, 255.07	Isoliquiritin
(5)	4.287	549.16, 531.15, 483.13, 429.10, 393.10	547.15, 529.15, 487.13, 457.12, 427.12, 367.08, 337.07	Chrysin-6-C-glu-8-C-ara
(6)	6.974	477.11, 499.07, 301.14	475.09, 299.06	Trihydroxy-methoxy-glu acid flavone
(7)	7.612	551.18, 419.14, 257.08	517.14, 255.07	Isoliquiritin apioside
(8)	7.914	447.09, 469.08, 271.06	445.08, 269.05	Baicalin^*∗*^
(9)	8.788	449.11, 273.08	447.09, 271.06	5,6-dihydroxy-7-*O*-glu acid flavanone
(10)	9.124	447.09, 469.07, 271.06	445.11, 269.04	Baicalein-5-O-glu acid
(11)	9.997	431.10, 255.06	429.08, 253.05	Chrysin-glu acid
(12)	10.198	461.11, 285.08	459.09, 283.06, 175.02, 113.02	Oroxylin A-7-*O*-glu acid
(13)	10.248	477.11, 301.07	475.09, 299.06	Trihydroxy-methoxy-glu acid flavone
(14)	10.266	461.11, 285.08	459.10, 919.19, 283.06, 268.04	Wogonoside
(15)	11.475	-	489.10, 13.07	Dihydroxy-dimethoxy-glu acid flavone
(16)	11.743	271.06, 253.05, 169.01	269.05, 197.06	Isowogonin
(17)	13.087	301.07, 286.05, 184.00	299.06	Trihydroxy-methoxyflavone
(18)	13.356	271.06, 253.05, 169.01	269.05	Baicalein^*∗*^
(19)	14.572	331.08	329.07	Trihydroxy-dimethoxyflavone
(20)	14.766	285.08, 270.05	283.06	Wogonin
(21)	17.857	285.08, 270.05, 168.01	283.06, 268.04	Oroxylin A
(22)	18.159	823.42, 647.38, 453.34	821.40	Glycyrrhizic acid^*∗*^
(23)	18.629	803.46, 763.47, 745.46, 619.42, 601.41, 455.36, 437.35	825.47, 799.46	Saikosaponin A^*∗*^
(24)	18.999	803.46, 763.47, 745.46, 619.42, 601.41, 455.36, 437.35	825.47, 799.46	Saikosaponin B_1_

ESI, electrospray ionization source; MS, mass spectrometry; glu acid, glucuronic acid. ^*∗*^Identified with reference standards.

**Table 10:** The prototype components and metabolites detected from plasma of mice after administration of MXCHT's *n*-BuOH extract.

Number	*t*_*R*_/min	MS data in ESI^+^	MS data in ESI^−^	Identification
M1	4.014	433.12, 257.08	431.10, 255.06	Liquiritigenin-glu acid
M2	4.216	433.11, 257.08	431.10, 255.07	Liquiritigenin-glu acid
P1	4.283	549.16, 531.15, 483.13, 429.10, 393.10	547.15, 457.12, 427.12, 367.08, 337.07	Chrysin-6-C-glu-8-C-ara
M3	5.878	-	621.11, 445.08, 269.04	Baicalein-diglu acid
M4	6.164	637.14, 461.11, 285.08	635.12, 459.10, 283.06, 268.03	Wogonin-diglu acid
M5	8.515	433.11, 257.08	431.10, 255.06	Isoliquiritigenin-glu acid
M6	8.985	461.11, 285.07	459.09, 283.06, 268.04	Wogonin-5-O-glu acid
P2	9.267	461.12, 483.09, 285.08	459.10, 283.06, 113.02	Oroxylin A-7-*O*-glu acid
P3	9.926	-	445.08, 269.04	Baicalein-5-*O*-glu acid
P4	9.993	-	429.08, 253.05	Chrysin-glu acid
P5	10.161	477.11, 301.07	475.09, 299.06, 284.03	Trihydroxy-methoxy-glu acid flavone
P6	11.484	461.11, 483.09, 285.08	459.10, 283.06	Wogonoside

ESI, electrospray ionization source; MS, mass spectrometry; P, prototype components; M, metabolites; glu acid, glucuronic acid.
